# Neutropenic Enterocolitis and Rapid Spontaneous Resolution of Portal Venous Gas: A Non-Respiratory Manifestation of COVID-19

**DOI:** 10.7759/cureus.9445

**Published:** 2020-07-28

**Authors:** Mahin Rehman, Amlish Gondal, Salman Khan, Najeeb U Rehman, Jaime Molina

**Affiliations:** 1 Internal Medicine, Guthrie Robert Packer Hospital, Sayre, USA; 2 Pulmonary and Critical Care Medicine, Geisinger Medical Center, Danville, USA; 3 Cardiology, Guthrie Robert Packer Hospital, Sayre, USA; 4 Critical Care Medicine, Guthrie Robert Packer Hospital, Sayre, USA

**Keywords:** covid-19 pandemic, covid-19, sars-cov-2, neutropenic enterocolitis, chemotherapy, gastroenterology, enterocolitis, infectious disease, immunosuppression, immunocompromised

## Abstract

The COVID-19 pandemic is affecting millions across the globe. The population of immunosuppressed individuals are at greatest risk of morbidity and mortality. Data on COVID-19 induced illness in the immunocompromised host are sparse. We aim to highlight the possibility of atypical and non-respiratory presentations of COVID-19 (severe acute respiratory syndrome coronavirus 2, SARS-CoV-2) in immunosuppressed individuals as our case reveals a rare COVID-19 associated GI presentation of neutropenic enterocolitis with bloody diarrhea.

## Introduction

The spectrum of the COVID-19 related disease manifestations is growing slowly. Primarily, the disease presents with a febrile illness and ultimately the development of respiratory symptoms with patients experiencing shortness of breath, tachypnea, and some decompensating into complete respiratory failure secondary to acute respiratory distress syndrome (ARDS) [1,2]. Aside from respiratory involvement, the disease has been linked to a thromboembolic state allowing individuals to have strokes and pulmonary emboli; it has also been linked to inducing myocardial injury whether in the form of myocarditis or myocardial infarction and has been linked to involvement of the gastrointestinal (GI) system [1,3-5].

COVID-19 is caused by the novel coronavirus severe acute respiratory syndrome coronavirus 2 (SARS-CoV-2), and it is a single-stranded ribonucleic acid (RNA) virus with notable spike proteins [2]. The natural animal reservoir of this virus seems to be the chrysanthemum bat and this virus is highly virulent and easily transmissible via droplets, direct and indirect contact, and possibly airborne transmission [2]. The virus enters the cell through angiotensin-converting enzyme 2 receptors (ACE2), which is expressed in the lungs, heart, vessels, and GI tract [2-5]. The spike proteins facilitate entry into cells by binding to the ACE2 receptors [2]. The subsequent infection can lead to a cytokine release storm due to the imbalance of T-cell activation with dysregulated release of cytokines, such as interleukin (IL)-6, IL-17, etc., leading to a severe inflammatory response [2-4]. Recent data have revealed that severe cases of COVID-19 are associated with proinflammatory immune pathways and those patients who are in critical condition in the ICU due to COVID-19 had significantly higher concentrations of certain cytokines; additionally, the infection was noted to induce leucopenia and lymphopenia [6].

There have been reports of GI involvement, though significantly less compared to respiratory and cardiac compromise, with regards to mild symptoms. With regards to severe involvement, there are very limited cases such as hemorrhagic colitis and severe acute pancreatitis [7,8]. However, there are no reports of severe GI cases of COVID-19 in the immunosuppressed host.

## Case presentation

A 72-year-old male with a past medical history of poorly differentiated adenocarcinoma of the lower esophagus and gastroesophageal junction (stage III), type II diabetes, hypertension, hyperlipidemia, coronary artery disease, and atrial fibrillation on rivaroxaban is undergoing cancer treatment with a curative intent via neoadjuvant chemoradiation (carboplatin and taxol) weekly; his most recent chemotherapy treatment was one week prior to admission into the hospital, where he presented with nausea, vomiting, and diarrhea. He is found to be hypotensive (blood pressure 70/30 mmHg) and febrile (104 degrees Fahrenheit). 

**Figure 1:**
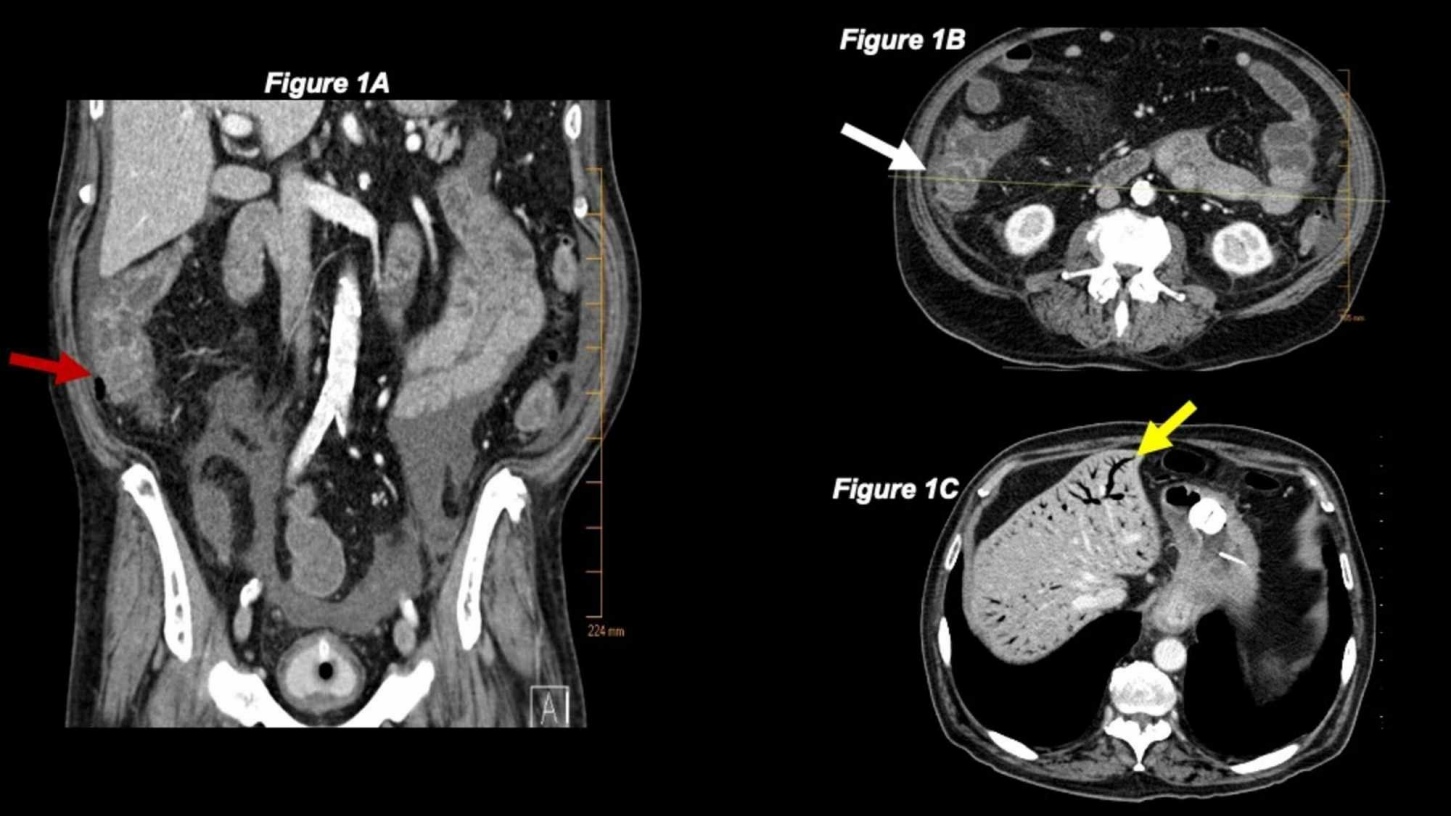
CT of the abdomen revealing the site of neutropenic enterocolitis and portal venous gas (A) Coronal view of CT abdomen showing the site of neutropenic enterocolitis and gas in paracolic gutter (red arrow). (B) Transverse view of CT abdomen showing the site of neutropenic enterocolitis (white arrow). (C) Transverse view of CT abdomen showing significant portal venous gas that eventually spontaneously resolved within hours on repeat CT abdomen (yellow arrow).

Lab work revealed him to be pancytopenic and his absolute neutrophil count (ANC) was low at 1.64 K/µL (reference range 1.8-7.7 K/µL). Physical exam revealed clear breath sounds bilaterally and mild abdominal tenderness in the right upper and lower quadrants with minimal distension. CT angiography of the chest, abdomen, and pelvis revealed a significant amount of new portal venous gas with abnormal mucosal enhancement and edema related to the distal ileum, cecum, and ascending colon in conjunction with air seen within the mesenteric vessels, and no thrombosis was noted (Figure 1). This was highly concerning for bowel ischemia but an infectious etiology, such as neutropenic enterocolitis or typhlitis, was on the differential. Both the CT scan and chest x-ray endorsed clear lung fields with no acute cardiopulmonary pathologies described in COVID-19 infections. His bowel movements were frankly bloody, so prothrombin complex concentrate and fresh frozen plasma were administered to reverse the effects of rivaroxaban. His abdominal pain and distension worsened, and surgery was deemed necessary.

Prior to surgery, SARS-CoV-2 RNA testing via nasopharyngeal swab was required, and he tested positive. Repeat CT scan prior to surgery was done to assess for mesenteric ischemia, and it showed spontaneous resolution of portal venous gas within hours of his initial CT; CT was negative for any mesenteric ischemia. His lab work was typical of an individual with an active COVID-19 infection: D-dimer >20 mcg/mL (reference range <0.5 mcg/mL), C-reactive protein 21.7 mg/dL (reference range < 1 mg/dL), ferritin 12,200 ng/mL (reference range 18-464 ng/mL), and lactate dehydrogenase 1,498 U/L (reference range 313-618 U/L) [6,9]. With an elevated procalcitonin of 5.69 ng/mL (reference range <0.012 ng/mL), a superimposed bacterial infection was suspected. The exploratory laparotomy showed a 90 cm long thickened ileus, consistent with neutropenic enterocolitis, and no evidence or signs of perforation, free air, bowel ischemia, obstruction, or purulent peritonitis were identified; the cause and source of gas noted on CT in the right paracolic gutter could not be identified. His bowels were deemed viable by the surgical team and resection was not performed. Blood cultures and stool studies including *Clostridium difficile* were negative; our institution did not have SARS-CoV-2 fecal testing. He failed to improve with IV broad-spectrum antibiotics, which is typically utilized in neutropenic patients with fever of unknown origin. Although neutropenic entercolitis allows bacteria to invade through the damaged gut wall because the host cannot mount an immune response against this, his failure to improve with antibiotics, despite being a non-specific sign, and worsening status made COVID-19 the most likely precipitating cause [10]. 

## Discussion

COVID-19 manifests primarily as a respiratory disease although many infected individuals can be asymptomatic. Other manifestations have been reported that coincide with respiratory involvement, such as strokes, pulmonary embolisms, and myocardial injury associated with febrile illness and respiratory issues. GI symptoms occur in less than 10% of patients who are infected with COVID-19 [11]. There have been very limited reports on bloody diarrhea and hemorrhagic colitis associated with COVID-19 [7]. This case of COVID-19 induced neutropenic enterocolitis with bloody diarrhea in an immunosuppressed host without respiratory involvement makes this a novel presentation. 

Studies show that neutropenic enterocolitis usually develops with an ANC < 500/µL and it is extremely rare to develop this with an ANC > 1,000/µL, which is another unique feature of COVID-19 induced enterocolitis [12,13]. To add to the rarity of this presentation, this individual presented with extensive portal venous gas concerning for bowel ischemia which managed spontaneously and rapidly (within a few hours) resolve on its own prior to being rule out via surgical exploration. He was managed conservatively postop and was successfully extubated one day after surgery to room air. 

There are a couple of limitations to note in this case report. Firstly, our institution did not have the capability for fecal SARS-CoV-2 RNA testing to definitively confirm the presence of the virus in the gut. Secondly, chemotherapy itself can induce mucosal injury (mucositis) and lead possible distension and necrosis along with gut dysmotility and ileus; though it is extremely unlikely for chemotherapy itself to cause neutropenic enterocolitis directly without pathogenic involvement of microorganisms as bacterial translocation is common, it must still be considered [12]. Lastly, given that his bowels were deemed viable, no intraoperative biopsy or resection was performed so histological analysis of the bowel was not able to be conducted for definitive confirmation.

## Conclusions

This case aims to bring light to physicians during this pandemic that not only are immunosuppressed individuals at greatest risk of morbidity and mortality, but that atypical and non-respiratory manifestations of COVID-19 in immunosuppressed individuals are indeed plausible. GI manifestations can occur in those infected with the SARS-CoV-2 virus as the GI tract does express the ACE2 receptor. 
